# Computational Approach to Identifying New Chemical Entities as Elastase Inhibitors with Potential Antiaging Effects

**DOI:** 10.3390/ijms252011174

**Published:** 2024-10-17

**Authors:** Giovanna Pitasi, Andrea Brancale, Sonia Floris, Antonella Fais, Rosaria Gitto, Laura De Luca

**Affiliations:** 1 Department of Chemical, Biological, Pharmaceutical and Environmental Sciences, University of Messina, Viale F. Stagno D’Alcontres 31, I-98125 Messina, Italy; giovanna.pitasi@studenti.unime.it (G.P.); ldeluca@unime.it (L.D.L.); 2 Department of Organic Chemistry, University of Chemistry and Technology, Prague, 166 28 Prague, Czech Republic; andrea.brancale@vscht.cz; 3 Department of Life and Environment Sciences, University of Cagliari, Monserrato, 09042 Cagliari, Italy; s.floris@unica.it (S.F.); fais@unica.it (A.F.)

**Keywords:** elastase, virtual screening, computational studies, *N*-substituted-1*H*-benzimidazol-2-yl]thio]acetamides

## Abstract

In the aging process, skin morphology might be affected by wrinkle formation due to the loss of elasticity and resilience of connective tissues linked to the cleavage of elastin by the enzymatic activity of elastase. Little information is available about the structural requirements to efficiently inhibit elastase 1 (EC 3.4.21.36) expressed in skin keratinocytes. In this study, a structure-based approach led to the identification to the pharmacophoric hypotheses that described the main structural requirements for binding to porcine pancreatic elastase as a valuable tool for the development of skin therapeutic agents due to its similarity with human elastase 1. The obtained models were subsequently refined through the application of computational alanine-scanning mutagenesis to evaluate the effect of single residues on the binding affinity and protein stability; in turn, molecular dynamic simulations were carried out; these procedures led to a simplified model bearing few essential features, enabling a reliable collection of chemical features for their interactions with elastase. Then, a virtual screening campaign on the in-house library of synthetic compounds led to the identification of a nonpeptide-based inhibitor (IC_50_ = 60.4 µM) belonging to the class of *N*-substituted-1*H*-benzimidazol-2-yl]thio]acetamides, which might be further exploited to obtain more efficient ligands of elastase for therapeutic applications.

## 1. Introduction

The skin plays the crucial role of acting as an effective barrier against the external environment in the human body. The skin is composed of different layers regulating distinct functions; in particular, the middle layer contains the connective tissue bearing an embedded fibroblast called dermis. It generates most of the skin properties through the so-called dermal extracellular matrix (dECM) that is rich in collagens, growth factors, elastic fibers, proteo- and glycosamino-glycans, and glycoproteins. Collagen and elastic fibers confer elasticity and resilience to the skin. Intrinsic and extrinsic factors generate skin aging processes through several physio-pathological pathways that induce specific structural and functional changes in the components of dECM, such as elastin (ELN) and collagen [1]. There is evidence that the main extrinsic factors might comprise the formation of reactive oxygen species (ROS) and peroxide species as secondary effect of excessive UV exposure. These radical species can affect the morphology of the skin, thus leading to wrinkle formation through a photo-aging process that can determine the sun-induced elastosis with loss of elasticity and resilience of connective tissue related to the proteolytic cleavage of ELN fibers suppressing the synthesis of dECM components [2,3,4]. Therefore, bioactive compounds with anti-elastase properties could delay skin aging processes and prevent sun-elastosis and, generally, skin aging [5]. The proteolytic activity of proteases is involved in various physiological and pathological processes [6], such as DNA replication, cell proliferation and death, and tissue remodeling, as well as cancer, neurodegeneration, and aging [7]. The degradation of collagen is regulated by Matrix metalloproteinases (MMPs) that are zinc-containing endopeptidases. Specifically, MMP-1 is considered responsible for collagen fragmentation during skin aging. On the other hand, the ELN structure and organization are regulated by the enzymatic activity of elastases that are serine proteases acting as endopeptidases; the elastases belong to the chymotrypsin family; eight genes encode for the elastase or elastase-like enzymes, four of which are classified as chymotrypsin-like. In humans, there are six elastase genes which encode elastase 1, 2, 2A, 2B, 3A and 3B. The Human Neutrophil Elastase (EC 3.4.21.37, HNE, elastase 2) plays an important role in the immune response towards bacterial infections, as well as tissue remodeling and inflammation [8,9]. Unlike other elastases, elastase 1 (EC 3.4.21.36) is expressed in skin keratinocytes [10].

Elastases target the hydrophobic protein ELN, which contains many repeated motifs of several alanine or valine aminoacidic residues punctuated by proline residues. It is well-known that serine proteases are classified based on their substrate specificity related to the kind of residue found at the S1 subsite as one of the distinct recognition sites for the polypeptide substrate [11]. The active site cleft of elastase comprises a catalytic triad (Ser, His, and Asp) combined with an oxyanion hole in which the backbone of Gly and Ser residues forms a positive-charged pocket activating the cleavage of the peptide bond. The S1 site hosting the corresponding P1 site of polypeptide substrate is a sub-pocket adjacent catalytic cleft; for elastase-like proteases, there are small hydrophobic residues at S1 such as Ala or Val. S1 is formed by residues 189–192, 214–216, and 224–228 [11]; the pocket specificity is generally related to the residues at positions 189, 216 and 226 [11]. Notably, the interaction between serine protease and polypeptide substrate is extended beyond the S1 site, involving additional binding pockets that influence the efficiency of the hydrolytic pathway [11].

Many efforts have been made to understand the binding mode of elastase inhibitors targeting the various isoforms in humans and other organisms; distinct structural information was collected through the analysis of the binding recognition in the similar target protein porcine pancreatic elastase (PPE) that was used as s cheaper enzyme for the preliminary biochemical assay. PPE has a high degree of sequence identity with pancreatic elastases from other species. The primary structure of PPE displays only 40% homology to that of Human Leukocyte Elastase (HLE); however, the tertiary structures of PPE and HLE are very similar in the active site area [12,13,14].

In more detail, PPE [13,15] comprises a single polypeptide chain of 240 amino acids [13,16] linked with four disulfide bridges. PPE is complexed with Ca^2+^ that is located in the so-called “Calcium binding site” near to catalytic cleft, and it is necessary for PPE stability. The polypeptide chain starts with Val16 and terminates with Asn245; this has been based on bovine chymotrypsinogen A numbering [17]; however, in the most consistent nomenclature, the canonical catalytic triad is composed by Ser203 (195), His60 (57) and Asp108 (102). PPE consists of four subsites S1, S2, S3, and S4 [10], which bind the acyl group side of the substrate, whereas the subsites S1′ and S2′ bind the leaving group during the catalytic cycle; a detailed description in the subsite composition of PPE was reported in the literature by Bode and coworkers [12].

The most popular and potent elastase inhibitors contain a peptide chemical scaffold mimicking the endogenous substrate, as observed for human protein elafin, that is physiologically produced in several tissues and exerts a protective effect from destruction by the up-regulated immune responses [18]. Additionally, several peptide- and nonpeptide-based inhibitors act as irreversible inhibitors, establishing covalent interactions with crucial residues located in the catalytic cleft. These compounds have been especially developed to fight pathological processes related to the over-activity of HNE.

**Figure 1:**
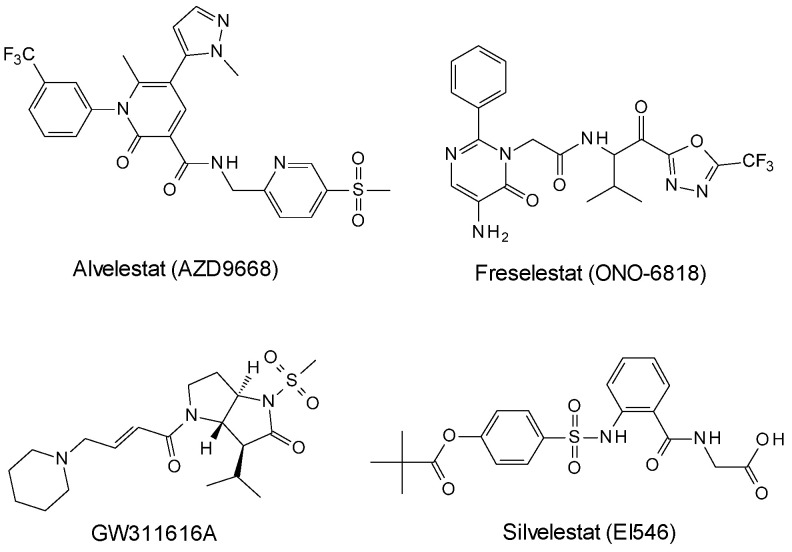
Non-peptide-based inhibitors of elastase that have reached (pre)clinical development.

The medicinal chemistry efforts to identify inhibitors against HNE provided a wide series of synthetic derivatives [19] as peptide and non-peptide substrate-based electrophilic ketone inhibitors; these compounds belong to the series of trifluoromethyl-based compounds such as the *N*-[[5-(methanesulfonyl)pyridin-2-yl]methyl]-6-methyl-5-(1-methyl-1*H*-pyrazol-5-yl)-2-oxo-1-[3-(trifluoromethyl)phenyl]-1,2-dihydropyridine-3-carboxamide (AZD9668, alvelestat, Figure 1) [20]; in addition, other non-peptide compounds possess distinct electron-withdrawing moieties as found in the 5-amino-N-[1-[[5-(1,1-dimethylethyl)-1,3,4-oxadiazol-2-yl]carbonyl]-2-methylpropyl]-6-oxo-2-phenyl-1(6H)-pyrimidineacetamide (ONO 6818, also known as freselestat, Figure 1) investigated for pulmonary diseases [21,22]. Moreover, it has been developed the inhibitor (3S,3aS,6aR)-3-isopropyl-1-(methylsulfonyl)-4-((E)-4-(piperidin-1-yl)but-2-enoyl)hexahydropyrrolo [3,2-b]pyrrol-2(1H)-one (GW311616A, Figure 1) possessing a trans-lactam substructure for which the mechanism of action has been elucidated through the analysis of its crystal complex with PPE (PDB 1HV7, vide infra) [23]. In March 2020, the inhibitor 2-[[2-[[4-(2,2-dimethylpropanoyloxy)phenyl]sulfonylamino]benzoyl]amino]acetic acid (Sivelestat, Figure 1) was approved for the treatment of inflammatory syndromes acting as a competitive inhibitor of NE [24]. Furthermore, several natural-based compounds containing phenolic moieties were demonstrated to inhibit elastases [25,26,27].

Apart from the well-established role of elastase 2 in inflammatory processes, there is evidence that the other elastases could offer the opportunity to develop active agents in human diseases. Recently, we focused our interest on developing new chemical entities that can prevent skin pathologies related to excessive melanogenesis related to skin pathologies. In detail, we identified tyrosinase inhibitors [27,28,29,30,31,32,33,34] to ascertain the role of tyrosinase inhibition in the development of potential dermatological agents like thiamidol.

Keeping in mind the crucial role of ELN inhibition in controlling the skin elasticity, in this work, we developed a computational approach to identify further non-peptide-based molecular entities for the development of elastase inhibitors able to prevent pathological conditions leading to sagging and wrinkling skin. We focused our interest on small molecules based on the consideration that they generally display more favorable physiochemical properties and are more prone to further structural optimization. Herein, we report a multistep and combined computational protocol enabling the development of an optimized pharmacophore model for elastase inhibitors; the model was subsequently applied to screen out our in-house database of compounds from the synthetic source. Then, the outcomes of the virtual screening procedure were filtered off to further select the most affordable compounds that were preliminarily tested against the PPE assay.

## 2. Results

### 2.1. Pharmacophore Modeling Generation

**Figure 2:**
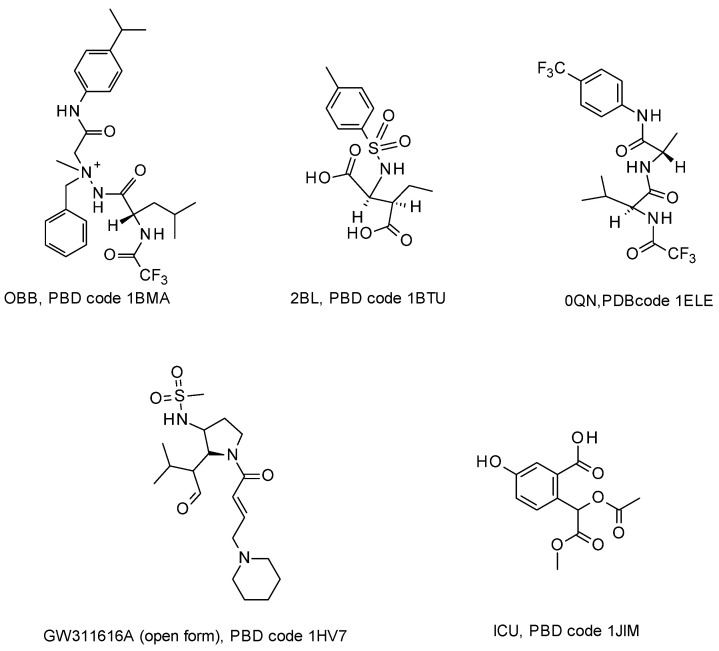
Chemical structures of the five ligands bound to PPE in the selected ligand/protein complexes available on PDB database (PDB codes: 1 BMA, 1 BTU, 1 ELE, 1 HV7, 1 JIM) [23,35,36,37,38].

To gain knowledge on the binding requirements to target the protein of interest, a structure-based pharmacophore modeling of pancreatic elastase-inhibitor complexes was conducted. Among all the available X-ray structures of PPE-inhibitor complexes, we selected five complexes (PDB codes: 1 BMA, 1 BTU, 1 ELE, 1 HV7, 1 JIM) [23,35,36,37,38] based on resolution and R-factor values (see experimental methods). The chemical structures of the ligands in complexes with the PPE are reported in Figure 2.

**Figure 3:**
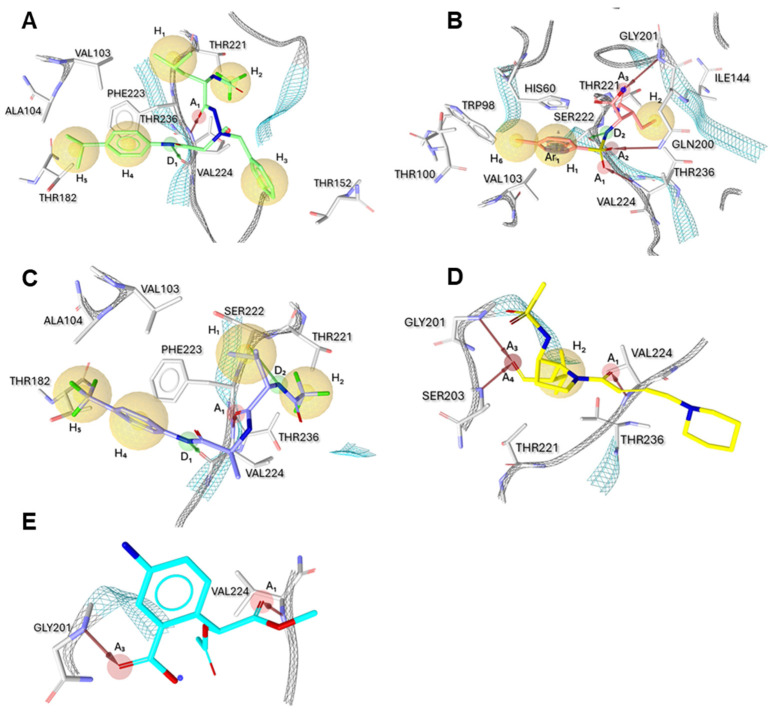
(**A**–**E**). The 3D structure-based pharmacophore models of PPE bound to different inhibitors derived from X-ray structure of complexes with PDB ID: (**A**) 1 BMA, (**B**) 1 BTU, (**C**) 1 ELE, (**D**) 1 HV7 and (**E**) 1 JIM (from references [23,35,36,37,38]). Target amino acids are shown as gray-colored sticks. Hydrophobic features are shown as yellow spheres, while hydrogen bond acceptors and donors are represented as red and green arrows, respectively.

Figure 3A–E describe the five distinct models obtained by LigandScout (version 3.12) [39], which is a platform to build three-dimensional pharmacophore models starting from protein/ligand complexes available in the archive PDB database. For each PDB entry, LigandScout generated a ligand-based hypothesis that was defined by a set of chemical features that were consistent with the aminoacidic residues surrounding each ligand bound to PPE.

The five pharmacophore models can be described as follows.

(1) Figure 3A illustrates the pharmacophore extracted from PDB code 1 BMA for the complex with an aminimide-based peptidomimetic inhibitor 0BB; this model consists of five hydrophobic features (H_1_–H_5_ yellow spheres): H_1_ given by the interaction with Val103; H_2_ corresponding to the interactions with residues Thr221, Val224, Thr236; H_3_ with Thr152; H_4_ by interaction with Val103 and Phe223; and H_5_ given by interaction with Val103, Ala104, Thr182 and Phe223. Additionally, it is described the interaction with the main chain (backbone) of Val224 created both hydrogen bond acceptor feature (A_1_, red arrow) and one hydrogen bond donor feature (D_1_, green arrow).
(2) In Figure 3B, it is reported that the pharmacophore extracted from 1 BTU for the complex with the (3*R*)-3-ethyl-N-[(4-methylphenyl)sulfonyl]-L-aspartic acid (2 BL) is derived as an acyl−enzyme complex formed between PPE and the monocyclic β-lactam-based inhibitor. For this second model, three hydrophobic features were generated: specifically, H_1_ for the interaction with Val103; H_2_ given by the interaction with four residues Ile144, Thr221, Val224 and Thr236; H_6_ for interaction with Trp98, Thr100, and Val103. The model comprises three hydrogen bond acceptor interaction features related to Gln200 (A_2_), Gly201 (A_3_), and Val224 (A_1_). Finally, one hydrogen bond donor feature for the interaction with Ser222 (D_2_) and one hydrophobic aromatic feature for interaction with crucial residue His60 (Ar_1_) are defined.
(3) The pharmacophore extracted from the ligand–protein complex 1 ELE is represented in Figure 3C. In this case, the residue Val103 corresponds to the hydrophobic feature (H_1_), whereas the hydrophobic feature H_2_ is generated by the interaction with residues Thr221, Val224, and Thr236; Val 103 and Phe223 are responsible for the hydrophobic feature H_4_; finally, Val103, Ala104 and Thr182 give (H_5_). The pharmacophore also presents one hydrogen bond acceptor feature (A_1_) corresponding to residue Val224. Finally, two hydrogen bond donor features are defined by interaction with Ser222 (D_2_) and Val224 (D_1_).
(4) Figure 3D shows the pharmacophore extracted from the 1HV7 containing the trans-lactam, GW311616A (see Figure 1); the bound represents the opened form of the inhibitor that allows us to define one hydrophobic feature (H_2_) given by the interaction with residues Thr221, Val224 and Thr236; additionally, the model consists of three hydrogen bond acceptor features created by residues Gly201 (A_3_), Ser203 (A_4_) and Val224 (A_1_).
(5) The last model was created from the complex 1 JIM (Figure 3E) for the ligand methyl(2-acetoxy-2-(2-carboxy-4-amino-phenyl))acetate (ICU). This model is composed of two simple hydrogen bond acceptor features generated by Gly201 (A_3_) and Val224 (A_1_).

All the pharmacophores possessed various excluded volumes that reflected potential steric restrictions and corresponded to areas where the ligand was not able to be localized. The pharmacophore model construction established many crucial contacts between protein and inhibitors. Overall, sixteen amino acid residues of PPE were involved; among them, several residues belonged to the four sub-pockets (S1, S2, S3, and S4) as well as from crucial catalytic triad and oxyanion hole. In particular, the selected residues were as follows: His60 (S2 and triad), Trp98, Thr100, Val103 (S2), Ala104 (S4), Ile144 (S1), Thr152, Thr182 (S4), Gln200 (S_1_), Gly201 (oxyanion hole), Ser203 (triad and oxyanion hole), Thr221 (S1), Ser222, Phe223 (S2), Val224 (S1, S3) and Thr236 (S1).

### 2.2. Contribution of Amino Acid Residues to Binding Free Energy

To improve the reliability of the five derived structure-based pharmacophore models, we carried out an in silico alanine scanning mutations [40]; we investigated the role of selected aminoacidic residues to the binding free energy for the five studied PDB structures (1BMA, 1BTU, 1ELE, 1HV7, 1JIM) [23,35,36,37,38].

**Figure 4:**
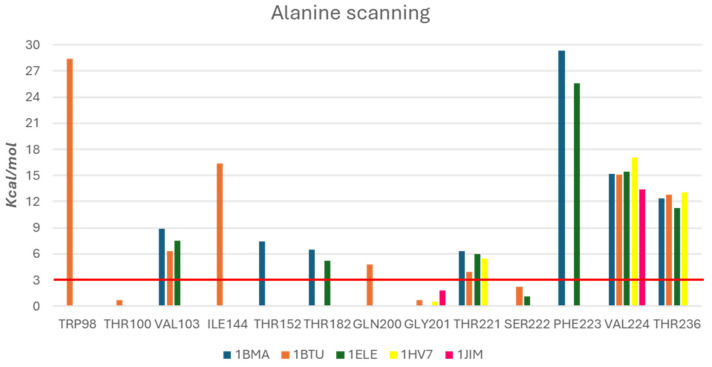
Schematic representation of the contributions of ΔΔG*_stability_* analyzed by using Alanine scanning module in Schrodinger. Each colored bar displays the interaction between each PDB structure and key amino acid residues. The red line represents the threshold of ΔΔG*_stability_* > 3 Kcal/mol.

The computational alanine scanning calculated the values of the binding free energy of the protein–ligand complex for selected amino acids before and after applying mutations to alanine. Consequently, the difference in binding free energy allowed us to obtain a quantitative measure of the free energy contribution of the specific residue to the binding free energy of the protein–ligand complex [40]. By analyzing the results, it might be possible to confirm how some mutations caused a weakening in protein–ligand interactions, as evidenced by the positive value of predicted Stability Change (ΔΔG*_stability_*.). The obtained ΔΔG*_stability_* values generated a ranking of the contribution to the binding, distinguishing the most significant hot spots (ΔΔG*_stability_* > 3 Kcal/mol) from the less relevant residues. This computational study was performed by using the Alanine scanning module in Schrödinger (Schrödinger Release 2023-2: Glide, Schrödinger, LLC, New York, NY, USA, 2021) [41], enabling the calculation of the energy contribute of a given amino acid to the total binding free energy for each protein–ligand complex. The analysis was conducted excluding the residue Ala104; moreover, we excluded residues His60 and Ser203 considering that these residues exerted an unambiguous role in catalytic activity of serine protease. The outcomes of this study were collected in Figure 4; for each PDB structure, one or more selected key residues were studied on the basis of previous data collected by pharmacophore hypothesis generation (see Section 2.1).

Notably, the mutation of residue Val224 caused a significant worsening of the binding free energy in all examined protein–ligand complexes. Moreover, mutations of Thr236 and Thr221 showed the worsening of binding free energy in four different structures examined. We decided to consider the amino acids with a ΔΔG*_stability_* > 3 Kcal/mol as relevant, paying more attention to residues Trp98, Val103, Thr221, Phe223, Val224 and Thr236, which appeared to be the key residues in most of studied ligand complexes [42,43] and possessed a high stability value.

### 2.3. Merged Pharmacophore Model

**Figure 5:**
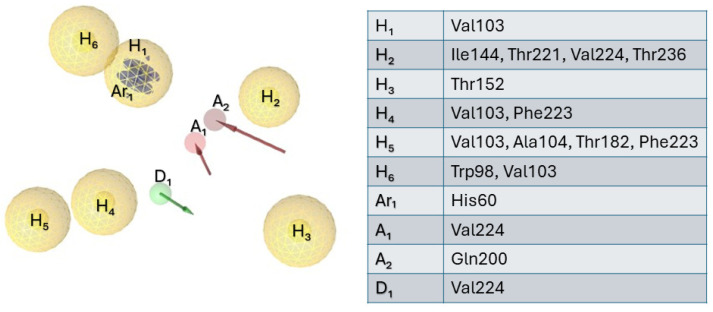
Merged pharmacophore composed of six hydrophobic features (yellow spheres, H_1_–H_6_), two hydrogen bond acceptors (red arrows, A_1,2_), one hydrogen bond donor (green arrow, D_1_), and one aromatic ring (Ar_1_). The table shows the label of the features and the corresponding amino acid residues involved in the interaction.

In this step of our computational protocol, the main suggestions extractable from the alanine-scanning study were employed to further refine the five obtained pharmacophore models (see Figure 3A–E); we especially focused our interested on five residues, Val103, Ala 104, Thr221, Val224 and Thr236; these residues lined the sub-pockets and exerted a fine tuning in elastase selectivity when compared to other serine proteases. Based on this consideration and merging the five pharmacophore models, we obtained the newer pharmacophore hypothesis that is depicted in Figure 5; this optimized hypothesis was composed of the following features: 6 hydrophobic features (H_1_–H_6_), 2 hydrogen bond acceptor features (A_1_–A_2_), 1 hydrogen bond donor feature (D_1_), 1 aromatic ring feature (Ar_1_), as well as 29 excluded volumes, that were leave out in the schematic representation displayed in Figure 5.

### 2.4. Molecular Dynamic Simulations

**Figure 6:**
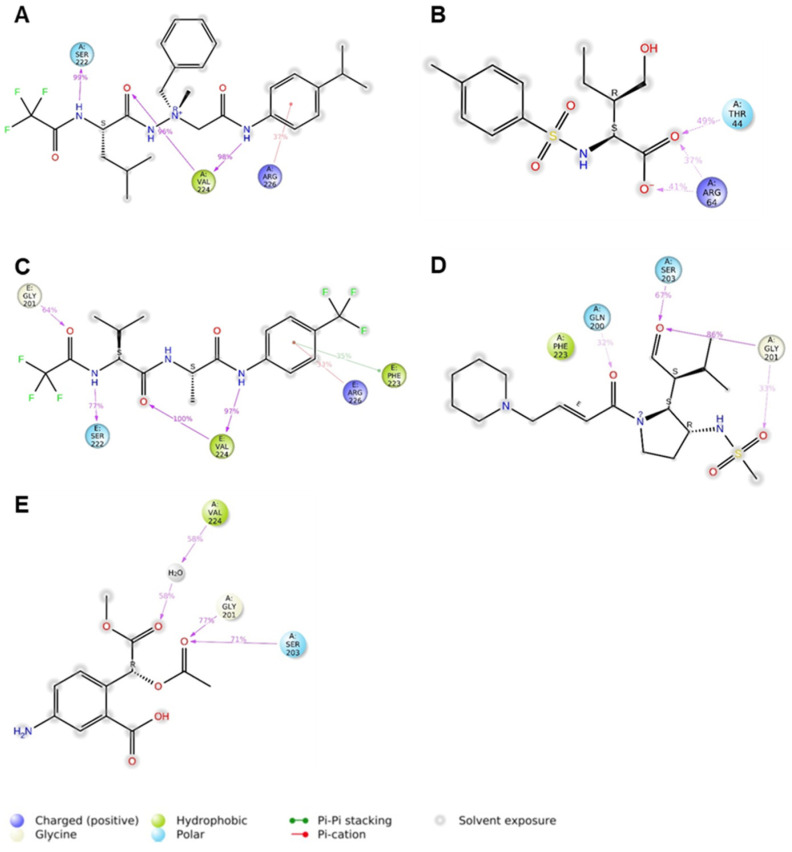
Molecular dynamics results of complexes of 1 BMA (**A**), 1 BTU (**B**), 1 ELE (**C**), 1 HV7 (**D**), and 1 JIM (**E**). Interactions that occurred for more than 30% of the simulation time were examined.

Considering that the high number of obtained features could be too strict to carry out a preliminary virtual screening, in this step of our computational study, we decided to further refine the model with the employment of additional calculations. We chose to perform molecular dynamic (MD) simulations to obtain more data on the crucial contacts, thus improving the chance to identify new promising compounds. The five PDB structures of elastase/ligands 1 BMA, 1 BTU, 1 ELE, 1 HV7 and 1 JIM(E), which were previously applied to create pharmacophore models (see Section 2.1), were now used to perform molecular dynamic studies by using Desmond tool in the Schrödinger software suite (Schrodinger 2023-2) [44,45]. By generating a root mean square deviation (RMSD) diagram for all MD simulations (see Table S1 in the Supplementary Materials), we verified the stability of protein–ligand complexes during the simulation. Figure 6 shows the results of the MD studies that revealed interactions occurring more than 30% of the simulation time. Therefore, we chose to consider relevant the following residues: Thr44, Arg64, Gln200, Gly201, Ser 203, Val221, Ser 222, Phe223, Val224 and Arg226. Cross-referencing these data with the outcomes of computational alanine scanning (see Section 2.3), we can assume that for the studied crystal complexes, the most relevant contacts might be summarized as follows: (i) polar interactions with Val 224 and Gln200; (ii) stable hydrophobic interactions with Phe223.

### 2.5. Simplified Pharmacophore Model

**Figure 7:**
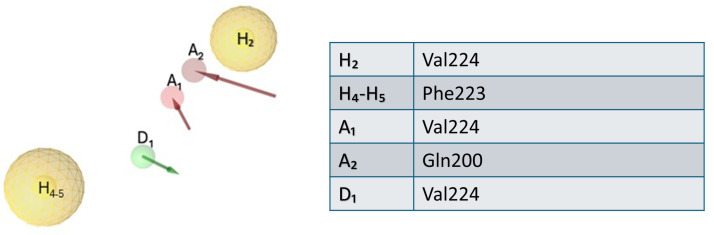
Refined pharmacophore model for elastase inhibitors composed of two hydrophobic features (yellow spheres, H_2_, H_4,5_), two hydrogen bond acceptor features (red arrows, A_1_–A_2_), one hydrogen bond donor (green arrow, D_1_). The table shows the label of the features and the corresponding amino acid residues involved in the interaction.

The results obtained from MD and alanine scanning studies enabled us to simplify our model. In more detail, we maintained the hydrogen bond features (A_1_, A_2_, and D_1_) considering the crucial contacts with Gln200 and Val224; a further refinement was carried out on the hydrophobic features as follows: we considered the hydrophobic interactions H_2_ given by Val224, as well as H_4_ and H_5_, important for the interaction with Phe223. Finally, considering that the two hydrophobic features H_4_ and H_5_ were very close to each other, we decided to incorporate them in one single feature increasing the tolerance. In turn, we discharged the redundant hydrophobic feature H_1_ as well as the poor relevant hydrophobic feature H_3_ while maintaining 29 excluded volumes. Now, the refined pharmacophoric hypothesis for elastase inhibitors consisted of five features that were related to the interactions with key residues revealed by combining the results reported in Figure 5 and Figure 6: two hydrophobic features (H_2_, H_4,5_, two hydrogen bond acceptor features (A_1_, A_2_), hydrogen bond donor feature (D_1_), as illustrated in Figure 7.

### 2.6. Virtual Screening and Biological Assay

**Figure 8:**
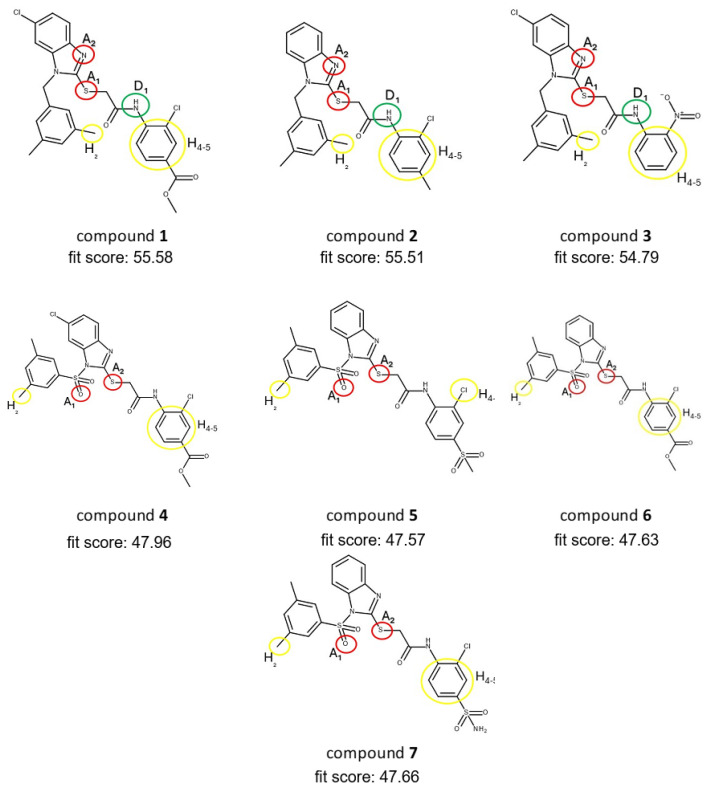
Mapping of the five chemical features 2 HY (H_2_, H_4,5_), 2 HBA (A_1_, A_2_), 1 HBD (D_1_) on *N*-substituted-1H-benzimidazol-2-yl]thio]acetamides (**1**–**7**) selected by virtual screening. Red circle for hydrogen bond acceptor feature; green circle for hydrogen bond donor feature; yellow circle for hydrophobic feature.

Based on the optimized 3D pharmacophore model displayed in Figure 7, we subsequently screened our in-house library of compounds named CHIME23, that consists of a large collection of small molecules bearing distinct heterocyclic scaffolds. Among them, the most populated chemical classes were quinolines, isoquinolines, imidazoles, triazoles, benzimidazoles, thiazoles, thiadiazoles, and 2,3-benzodiazepines. These compounds were synthesized in the medicinal chemistry laboratory by Laura De Luca, Rosaria Gitto, and their coworkers at the University of Messina during the last few decades. In more detail, CHIME23 collects small molecules that have been proved to be active ligands targeting various pathological pathways in human pathologies (antivirals, neuroprotective, anticonvulsants, and anticancers). Despite the large chemical space of compounds from the CHIME23 collection, the virtual screening query furnished hits belonging to the only class of *N*-substituted-1*H*-benzimidazol-2-yl]thio]acetamides that we have previously developed as anti-HIV-1 non-nucleoside reverse transcriptase inhibitors [46]. In particular, seven compounds **1**–**7** proved to possess the best pharmacophore fit scores and matched the identified chemical features, as detailed in Figure 8.

Considering that the selected compounds **1**–**7** might assume a very similar positioning in the active site of PPE, the selection criteria to perform the subsequent focused in vitro study included the evaluation of few structural variations: (i) the role of substituent on benzene-fused ring of the benzimidazole fragment; (ii) the presence of a single or a pair of substituents on aniline moiety; (iii) the impact on inhibitory effects of the nature of the linking group of 3,5-dimethylphenyl substituent. Moreover, we chose to exclude compounds **1**, **4** and **6** to avoid the non-specific esterase activity of PPE toward the ester moiety located at the para position of aniline moiety. Therefore, we selected the *N*-(2-chloro-4-methylphenyl)-2-[[1-[(3,5-dimethylphenyl)methyl]-1*H*-benzimidazol-2-yl]thio]acetamide (**2**), 2-[[6-chloro-1-[(3,5-dimethylphenyl)methyl]-1*H*-benzimidazol-2-yl]thio]-*N*-(2-nitrophenyl)acetamide (**3**), and *N*-(2-chloro-4-sulfamoylphenyl)-2-[[1-(3,5-dimethylbenzenesulfonyl)-1*H*-1,3-benzodiazol-2-yl]sulfanyl]acetamide (**7**) to carry out the experimental testing of elastase inhibition. None of the selected compounds possessed PAINS alerts as determined by SwissADME platform (www.swissadme.ch, accession date 12 May 2024). The preliminary biochemical assay was performed by using PPE, the substrate N-succ-(Ala)3-nitroanilide (SANA) and the standard compound oleanolic acid (IC_50_ = 25.7 ± 1.38 µM [47]). Unfortunately, compounds **3** and **7** failed to display ≥30% of the inhibitory threshold at fixed doses of 50 µM. On the other hand, compound **2** demonstrated a promising inhibitory effect toward PPE (IC_50_ = 60.4 ± 1.98 µM), suggesting that the *N*-substituted-1H-benzimidazol-2-yl]thio]acetamide chemotype could be further exploited to develop new chemical entities targeting elastase.

### 2.7. Molecular Docking

**Figure 9:**
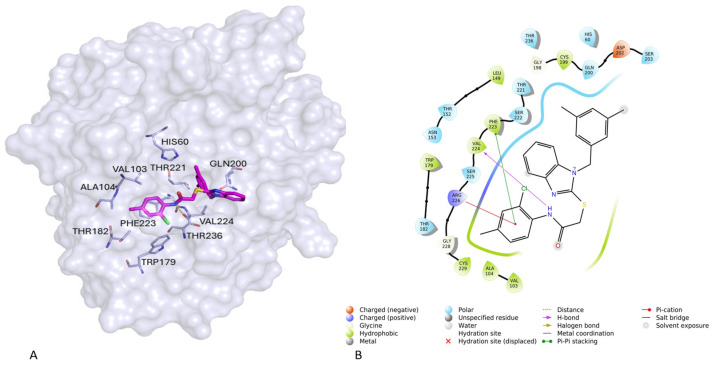
(**A**) Plausible binding mode of compound **2** (green stick) in the cavity of elastase protein structure (gray). The hydrogen bond is represented as yellow dashes. This figure was prepared using the program PyMOL (https://www.pymol.org accessed on 14 August 2024, The PyMOL Molecular Graphics System, Version 3.0 Schrödinger, LLC., New York, NY, USA). (**B**) Schematic 2D representation of the interactions between compound **2** and PPE, the interactions were generated by Maestro.

As revealed by several structural experiments, the active site of elastase is composed of distinct areas, including the crucial regions of the catalytic site/oxyanion hole that are surrounded by two sets of sub-pockets (S1–S4 and S1′–S3′), that anchor the substrate and subsequently generate the release of the leaving fragment. Therefore, the active site is an extended area enabling the accommodation of peptide- and non-peptide-based inhibitors. To gain information on the possible binding conformation of the most active compound **2** and its interaction mode with PPE, a flexible docking study was performed using the protein extracted from the structure of the complex elastase/0QN (PDB code 1ELE), using the program GOLD (v2024) [48]. The best docking conformation of inhibitor **2** bound to PPE is illustrated in Figure 9A–B.

Compound **2** exhibited hydrophobic interactions with different residues of the binding site; in particular, **2** projected its aniline fragment toward the cavity composed of residues Val103, Ala104, Trp179 and Thr182 from the S4 sub-pocket. In addition, the -NH group established the hydrogen bonding interaction with the residue Val224, in coherence with the pharmacophore model and alanine scanning analysis. Finally, the dimethyl-benzyl tail and the benzimidazole moiety of compound **2** gave interactions with crucial residues His60, Gln200, Thr221, Phe223, Val224 and Thr236, belonging to the S1-S2-S3 subsites. The favorable positioning of inhibitor **2** into the active site might account for its ability to reduce the PPE activity in our preliminary in vitro assay (*cfr* previous section). Notably, the docking results were consistent with the pharmacophore information. Based on this evidence, we considered that the robustness of our docking protocol that might be applied to further identify newer compounds as analog compounds inspired by **2**, as well as new chemical entities through screening campaigns from different databases of available compounds.

## 3. Materials and Methods

### 3.1. Molecular Modeling Studies

#### 3.1.1. Protein Preparation

To select the PDB complexes useful to create the pharmacophore model, a comprehensive study was conducted on different structures available on the RCSB PDB (https://www.rcsb.org, accessed on 30 January 2024). The resolution and R-factor were examined for each protein structure, specifically, a resolution less than 2.5 Å and R-factor less than 0.2. As a result, five structures of PPE in the complex with different inhibitors, deposited on RSCB PDB (PDB codes: 1 BMA, 1 BTU, 1 ELE, 1 HV7, 1 JIM), were chosen (Table 1) applying the similar binding mode exhibited for all five inhibitors towards the five studied binary complexes as selection criteria.

Water and cofactors were deleted from each structure by Maestro. Subsequently, we renumbered residues (Val16-Asn255) via VegaZZ [49] (only for PDB: 1 BTU, 1 JIM) to obtain the same amino acid sequence numbering in each structure. The protein of 1 BTU was used as reference to align the other structures using PyMOL. Finally, the protein structures were prepared by means of the Protein Preparation Wizard (Schrodinger 2023-2) using the default settings, with the exception of missing chains that were added using the program Prime.

#### 3.1.2. Pharmacophore Model Generation

The pharmacophore model was generated using LigandScout. A model was built for each of the five PPE structures in complex with different inhibitors. Each model was submitted to post-alanine scanning refinement, and only those features defined as a hotspot via alanine-scanning study were maintained. Subsequently, a merged model was obtained and subjected to additional post-molecular-dynamic refinement. The resulting refined model was subjected to a validation process. A set of 15 co-crystallized ligands to the PPE protein available on the RCSB PDB were selected as the validation set (PDB codes: 1B0E, 1E36, 1ELD, 1ELF, 1FZZ, 1INC, 1MMJ, 1QGF, 2V35, 3HGP, 4YM9, 6QEN, 7EST, 8EST and 9EST).

Virtual screening was performed with the following settings:

- Scoring function: Pharmacophore fit;
- Screening mode: Match all query features;
- Retrieval mode: Obtain best matching conformation;
- Omitted features: 1.

Out of the 15 ligands of the validation set, 11 ligands fitted the model (see Supplementary Materials, Table S2) [16,37,50,51,52,53,54,55,56,57,58], so this model was considered predictive.

#### 3.1.3. Molecular Dynamics

For the five protein–ligand complexes, molecular dynamics were carried out using Desmond tool in the Schrödinger software suite (Schrodinger 2023-2). The orthorhombic simulation box (size 10A × 10A × 10A) was prepared with the TIP3P water model. To make the total charge of the system neutral, Na^+^ and Cl^−^ ions were added, maintaining a salt concentration of 0.15M. By selecting the OPLS4 force field, the protein–ligand complex was prepared for molecular dynamic simulation. The simulation of each protein structure has been set to the NPT option to maintain a fixed number of particles, temperature, and pressure parameters. Temperature and pressure were set to 300 K and 1 atm, respectively. A total of five molecular dynamics simulations were performed for a time of 500 ns for each simulation. The results of the MD simulation were analyzed using the Simulation Interaction Diagram tool implemented in the Desmond package.

#### 3.1.4. Molecular Docking

To validate the molecular docking protocol, several studies were performed on various proteins. The best results were obtained through the use of flexible docking of GOLD using the crystal structure of PPE (PDB code 1 ELE). We chose 1ELE to carry out our molecular docking experiments considering the best RMSD value (0.5310 Å) calculated on the best docking pose of the inhibitor and its co-crystallized pose, when compared to the values obtained restoring the other four inhibitors for each corresponding PDB structure. The superimposition of the experimental and restored pose of inhibitor 0QN is described in Figure S1 of Supplementary Materials.

In this case, amino acids were rendered flexible within 6 Å of the centroid of coordinates x: −12.2628, y: 20.1692, z: 37.7359 obtained from the 5 co-crystallized ligands used for the generation of the pharmacophore model (1 BMA, 1 BTU, 1 ELE, 1 HV7, 1 JIM). The residues made flexible include the following: His60, Val103, Gln200, Ser203, Ser222, Phe223 and Val224. All ligands were subjected to 100 runs and a 10 Å box was evaluated. The “Allow early termination” option was unchecked and the CHEMPLP function was used. All ligands were previously prepared using the LigPrep tool implemented in Maestro, generating the possible ionization states at pH 7.0 ± 2.0 using Epik (Schrödinger Release 2024-3: Epik, Schrödinger, LLC, New York, NY, USA, 2024) [59]. The (best) pose was examined for the study of interactions with the target using Maestro (Schrödinger Release 2024-3: Maestro, Schrödinger, LLC, New York, NY, USA, 2024).

### 3.2. Elastase Inhibition Assay

Elastase inhibition was determined by using the substrate *N*-succ-(Ala)-3-nitroanilide (SANA), as previously described [47]. The assay was performed in 0.1 M Tris-HCl buffer (pH 8.0). PPE (3.3 µg/mL) was incubated with or without the compound for 20 min, and after incubation, the substrate (1.6 mM) was added. The release of p-nitroaniline during cleavage of the substrate SANA by the enzyme activity was monitored at 410 nm. The control was performed with DMSO, while oleanolic acid, tested in the same experimental conditions, was used as a positive control. All chemical reagents were obtained as pure commercial products from Sigma Chemical Co (St. Louis, MO, USA) and used without further purification.

## 4. Conclusions

In this study, we report our structure-based drug discovery efforts for identifying new chemical entities that inhibit PPE. The large surface of the active site of elastase is composed of several sub-pockets combined with the catalytic area of this class of serine protease, possessing hydrolytic activity for the peptide-based substrate. To better understand the binding mode of active inhibitor from synthetic source, we performed a multistep computational protocol that afforded a simple and optimized pharmacophore model highlighting the most relevant residues for binding recognition. We combined distinct computational tools to select the model that filter off promising chemotypes from a collection of small compounds from the synthetic source.

The best outcomes of this study consisted of the description of the best chemical features for elastase inhibition as well as the identification of a new candidate for further structural optimization and structural–activity relationship analysis. This method might allow the exploitation of additional molecular interactions with unexplored sub-pockets of elastase. The combination of our pharmacophore model and docking procedure could be furnished as a useful protocol to perform further virtual screening campaigns towards external three-dimensional collections of compounds from synthetic and natural sources to discover new elastase inhibitors.

## Figures and Tables

**Table 1:** Resolution and R-factor values for the five selected PDBs.

PDB Entry	Resolution	R-Factor
1 BMA	1.80 Å	0.192
1 BTU	1.60 Å	0.192
1 ELE	2.00 Å	0.171
1 HV7	1.70 Å	0.150
1 JIM	2.31 Å	0.153

## Data Availability

Data will be made available from the authors upon request.

## Supplementary Materials

The following supporting information can be downloaded at: https://www.mdpi.com/article/10.3390/ijms252011174/s1.
