# Supplementary material for: Computational Approach to Identifying New Chemical Entities as Elastase Inhibitors with Potential Antiaging Effects
Source: Int J Mol Sci. 2024 Oct 17;25(20):11174. doi: 10.3390/ijms252011174 (PMC11508974; doi:10.3390/ijms252011174)

Supplementary Materials

Computational approach to identify new chemical entities as elastase inhibitors endowed of potential antiaging effects

Giovanna Pitasi [1], Andrea Brancale [2], Sonia Floris [3], Antonella Fais [3], Rosaria Gitto[1,*], and Laura De Luca[1]

1 Department of Chemical, Biological, Pharmaceutical and Environmental Sciences, University of Messina, Viale F. Stagno D'Alcontres 31, Messina I-98125, Italy
2 Department of Organic Chemistry, University of Chemistry and Technology, Prague, 166 28 Prague, Czech Republic.
3 Department of Life and Environment Sciences, University of Cagliari, 09042, Monserrato, Cagliari, Italy
* Correspondence: rosaria.gitto@unime.it; 00390906766413

Table of contents

Table S1. RMSD plots of MD simulations of protein-ligand complexes available in RCSB PDB (PDB codes: 1BMA, 1BTU, 1ELE, 1HV7, 1JIM)

Table S2. List of co-ligands used as validation set that fit the pharmacophore model.

Figure S1. Superimposition of the best docking pose with the co-crystallized pose (PDB code: 1ELE).

Table S1. RMSD plots of MD simulations of protein-ligand complexes

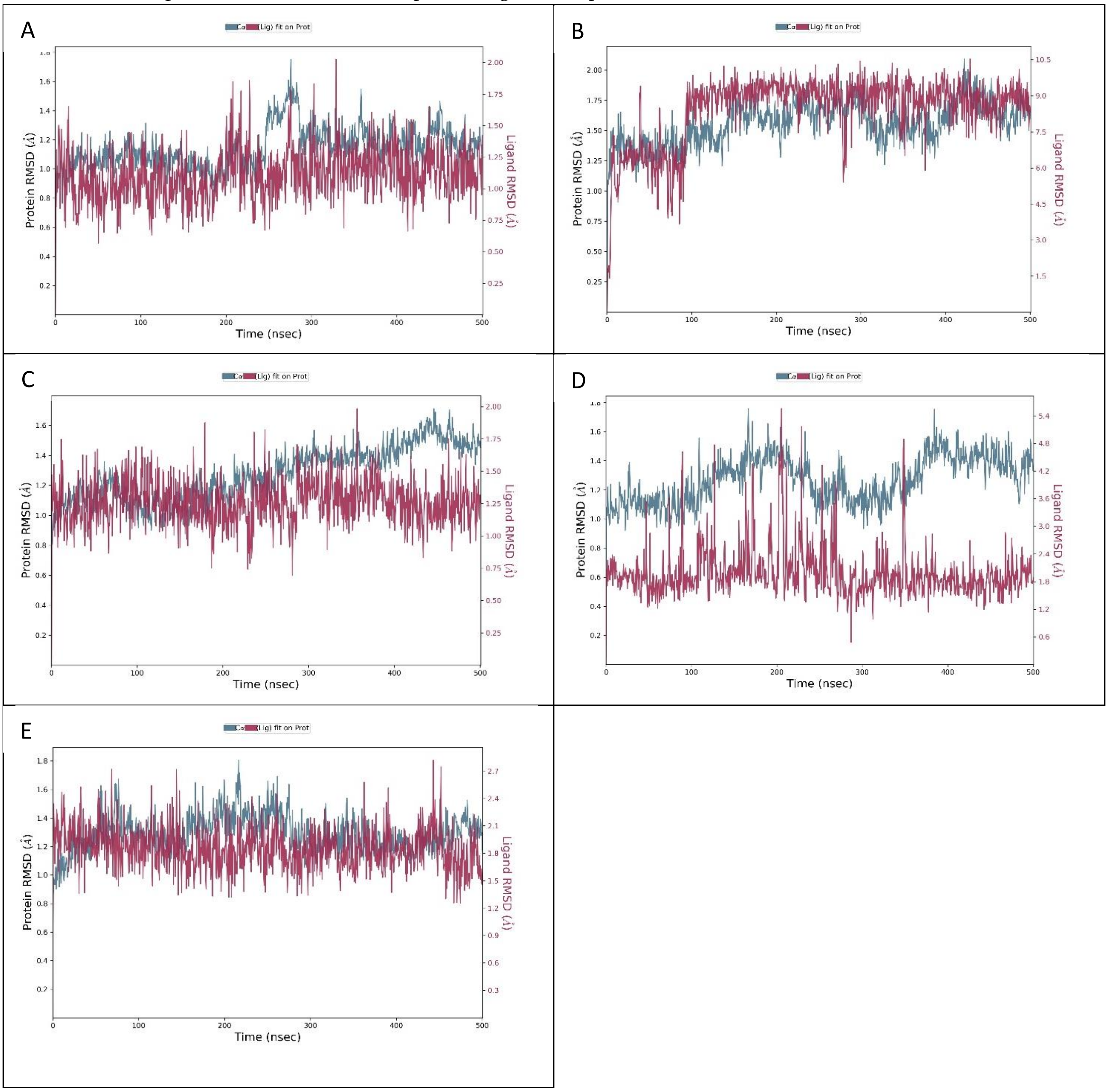

RMSD analysis of the selected complexes of porcine pancreatic elastase in complex with (A) ligand OBB (PDB code: 1BMA); (B) ligand 2BL (PDB code 1BTU); (C) ligand 0QN (PDB code 1ELE); (D) ligand GW311616A (PDB code 1HV7); (E) ligand ICU (PDB code 1JIM).

Table S2. List of co-ligands used as validation set that fit the pharmacophore model and relative pharmacophore fit-score value.

PDB	Reference/DOI	Figure	Fitscore
**2V25**	DOI: 10.1039/b706622h	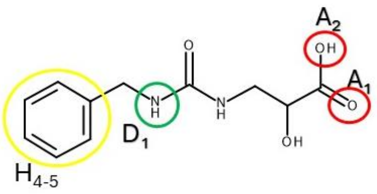	47,17
**1QGF**	DOI: 10.1021/bi990098y	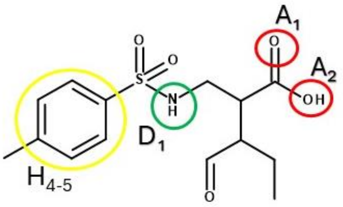	46,90
**1E36**	**PDB DOI:** 10.2210/pdb1E36/pdb	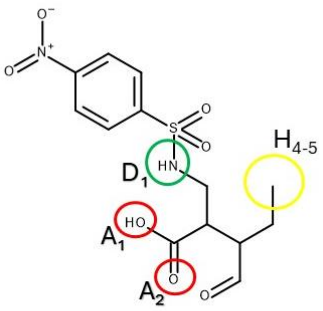	46,46
**1INC**	DOI: 10.1016/0022-2836(87)90291-9	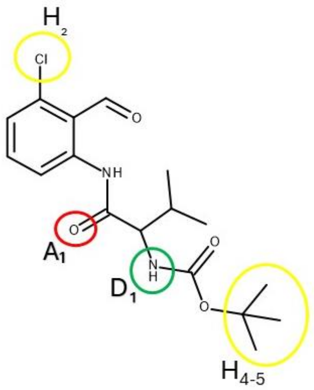	46,38

**7EST**	DOI: 10.1002/jmr.300030104	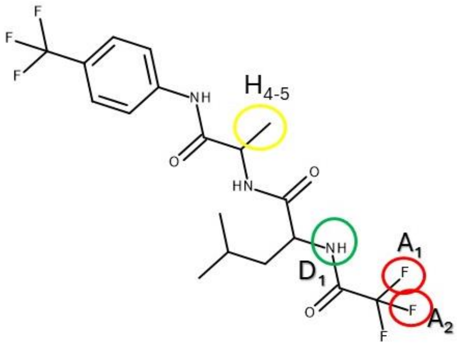	46,28
**1ELD**	DOI: 10.1021/bi00010a008	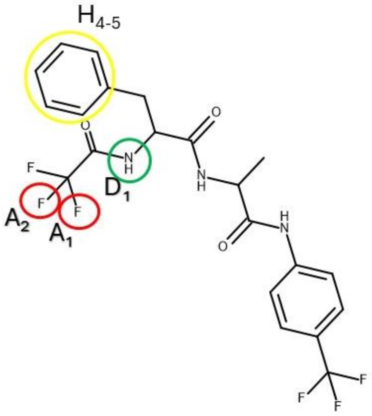	46,18
**4YM9**	DOI: 10.1002/cmdc.201600258	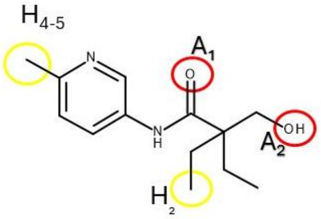	45,77
**3HGP**	DOI: 10.1021/ja9028846	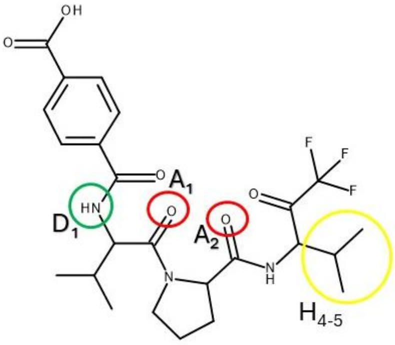	45,38

**1FZZ**	DOI: 10.1016/s0968-0896(00)00277-7	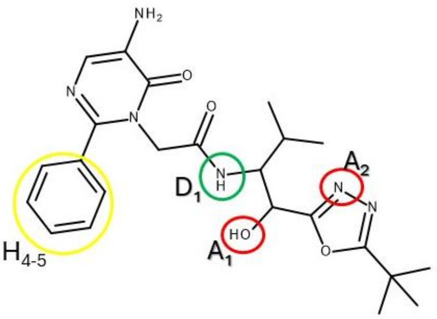	44,20
**1B0E**	DOI: 10.1021/jm970812e	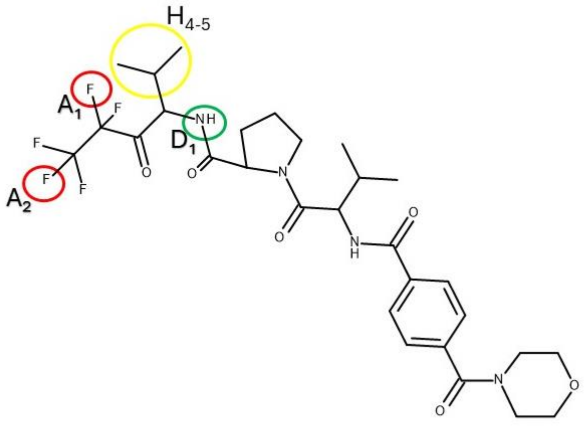	44,13
**6QEN**	DOI: 10.1021/acschembio.0c00090	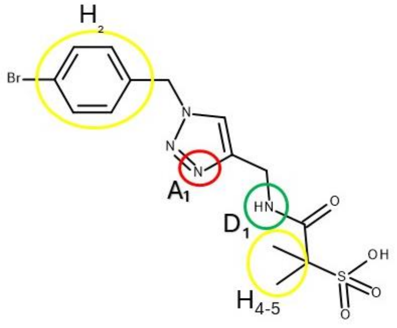	44,00

The validation set consisting of 15 ligands co-crystallized with porcine pancreatic elastase (PDB codes: 1B0E, 1E36, 1ELD, 1ELF, 1FZZ, 1INC, 1MMJ, 1QJF, 2V35, 3HGP, 4YM9, 6QEN, 7EST, 8EST, 9EST) was superimposed on the pharmacophore model. As result, the 11 co-ligands reported in Table S2 fitted the pharmacophore model.

Figure S1. Superimposition of the best docking pose of inhibiro with the co-crystallized pose (PDB code: 1ELE). Flexible docking studies were performed with GOLD on the 1ELE protein; this study resulted in a best pose if the inhibitor (yellow stick) that restored its co-crystallized pose (orange stick) with RMSD values of 0.53 Å. The figure was prepared using PYMOL software (The PyMOL Molecular Graphics System, Version 3.0 Schrödinger, LLC.)

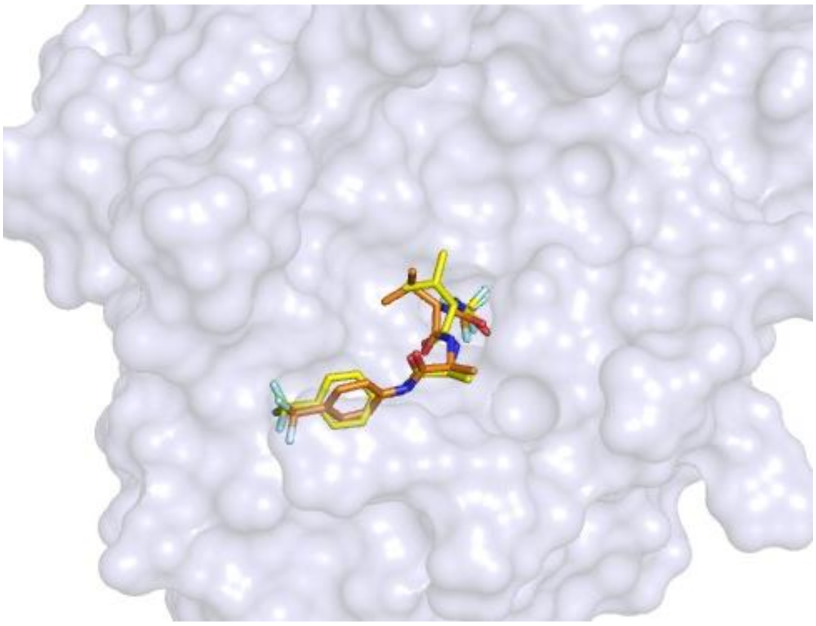

Supplement: Supplementary file 1 [file ijms-25-11174-s001.zip › ijms-3252181-supplementary.pdf]
